# Faecal DNA metabarcoding reveals novel bacterial community patterns of critically endangered Southern River Terrapin, *Batagur affinis*

**DOI:** 10.7717/peerj.12970

**Published:** 2022-03-29

**Authors:** Mohd Hairul Mohd Salleh, Yuzine Esa, Mohamad Syazwan Ngalimat, Pelf Nyok Chen

**Affiliations:** 1 Department of Aquaculture, Faculty of Agriculture, Universiti Putra Malaysia, Serdang, Selangor, Malaysia; 2 Royal Malaysian Customs Department, Presint 2, Putrajaya, Malaysia; 3 International Institute of Aquaculture and Aquatic Sciences, Universiti Putra Malaysia, Port Dickson, Negeri Sembilan, Malaysia; 4 Department of Microbiology, Faculty of Biotechnology and Biomolecular Sciences, Universiti Putra Malaysia, Serdang, Selangor, Malaysia; 5 Turtle Conservation Society of Malaysia, Kemaman, Terengganu, Malaysia

**Keywords:** Freshwater turtle, 16s rRNA, Peninsular Malaysia, V3–V4 region, Captive and wild, Taxonomic profile, Gut microbiota

## Abstract

Southern River Terrapin, *Batagur affinis*, is a freshwater turtle listed as critically endangered on the IUCN Red List since 2000. Many studies suggest that faecal DNA metabarcoding can shield light on the host-associated microbial communities that play important roles in host health. Thus, this study aimed to characterise and compare the faecal bacterial community between captive and wild *B. affinis* using metabarcoding approaches. A total of seven faeces samples were collected from captive (*N* = 5) and wild (*N* = 2) adult *B. affinis* aseptically, crossing the East and West coast of peninsular Malaysia. The DNA was extracted from the faeces samples, and the 16S rRNA gene (V3–V4 region) was amplified using polymerase chain reaction (PCR). The amplicon was further analysed using SILVA and DADA2 pipelines. In total, 297 bacterial communities taxonomic profile (phylum to genus) were determined. Three phyla were found in high abundance in all faeces samples, namely Firmicutes (38.69%), Bacteroidetes (24.52%), and Fusobacteria (6.95%). Proteobacteria were detected in all faeces samples (39.63%), except the wild sample, KBW3. Under genus level, *Cetobacterium*was found as the most abundant genus (67.79%), followed by *Bacteroides* (24.56%) and *Parabacteroides* (21.78%). The uncultured genus had the highest abundance (88.51%) even though not detected in the BK31 and KBW2 samples. The potential probiotic genera (75.00%) were discovered to be more dominant in *B. affinis* faeces samples. Results demonstrated that the captive *B. affinis* faeces samples have a greater bacterial variety and richness than wild *B. affinis* faeces samples. This study has established a starting point for future investigation of the gut microbiota of *B. affinis*.

## Introduction

As part of their ecological role, freshwater turtles maintain the health of enormous river grass beds. Their habitats support aquatic life, aid in maintaining healthy food webs in the water, and promote the transfer of nutrients from the river to terrestrial ecosystems (Bodie, 2001; Turtle Conservation Fund, 2002). Thus, they are regarded as important indicators of aquatic ecosystem health (Burke & , 1995; Browne & Hecnar, 2007). Unfortunately, as a result of human activities (Chen, 2017) (such as habitat destruction, river pollution, poaching, and fishing) (Chen, 2017) as well as climate change, the population of freshwater turtles has plummeted (Pike, 2013). As a result, the International Union for Conservation of Nature (IUCN) has classified 25 freshwater turtles as endangered (Stanford et al., 2020). Among them, the Southern River Terrapin, *Batagur affinis*, has been listed as critically endangered on the IUCN Red List since 2000 (IUCN, 2001).

**Figure 1:**
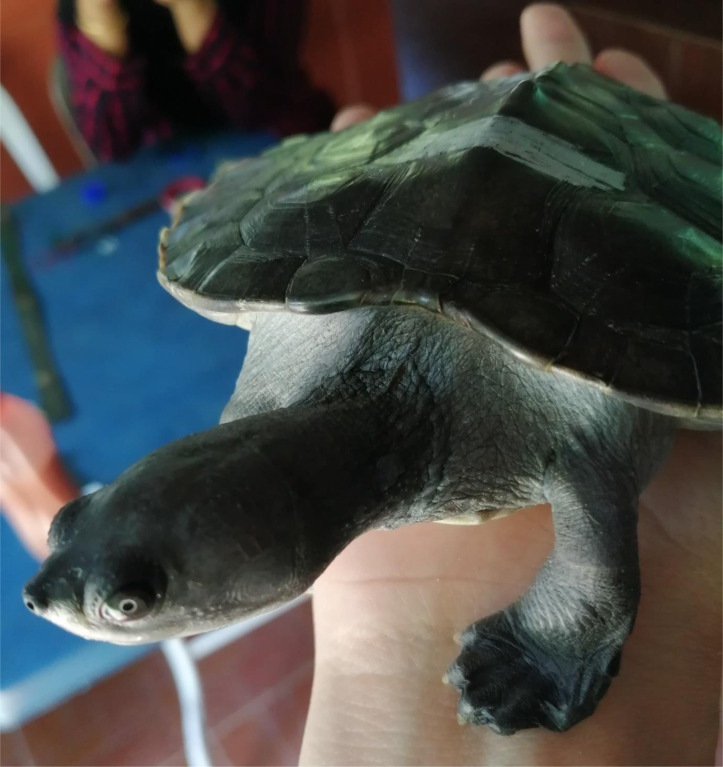
The subject matter in this study is the Southern River Terrapins (*Batagur affinis)* of Malaysia.

With advancements in molecular microbial community identification techniques (such as metabarcoding and metagenomics), the microbial community patterns and their potential roles related to the host’s health and disease can be determined. For instance, it has been found that the human gut microbial communities help facilitate metabolic and absorptive processes and stimulate immunity (Fujimura et al., 2010). Moreover, it has been suggested that symbiotic microbes in the frog, *Atelopus* sp., produce neurotoxin, tetrodotoxin, which protects the host from predators (Chau et al., 2011). In addition, microbial community studies in faeces samples using DNA metabarcoding technique have been reported as a non-invasive, accurate, and time-and cost-effective tool to determine host-associated microbial communities that play important roles in hosts’ health (Ando et al., 2020). Thus, due to the advances in molecular microbial community identification techniques, the exploration of the captive and wild *B. affinis* (Fig. 1) faeces samples in terms of bacterial community could enhance the understanding of gut microbiome patterns as their potential roles in *B. affinis*.

To date, scarcely any examinations have inspected freshwater turtle microbiomes, especially in *B. affinis*. Most investigations focus on sea turtles’ microbiomes (Ahasan et al., 2019; Biagi et al., 2019; Arizza et al., 2019). However, a plethora of research has suggested that faecal DNA metabarcoding can be an appealing way to deal with microbial communities (Ducotterd et al., 2021; Pompanon et al., 2012; Valentini, Pompanon & Taberlet, 2009). Also, this technique has been commonly used to study the diets of various animals (Ingala et al., 2021; Goldberg et al., 2020). One possible contributing factor of faecal DNA metabarcoding is in light of the symbiotic bacterial community patterns that might be useful for long-term conservation purposes of *B. afiinis*. Given the advantages of faecal DNA metabarcoding further investigation into the *B. affinis* gut microbiome is warranted.

The present study aimed to characterise and compare the faecal bacterial community between captive and wild *B. affinis* using metabarcoding approaches. The DNA was extracted from the faeces samples, and the 16S rRNA gene (V3–V4 region) was amplified using polymerase chain reaction (PCR). The obtained data were further analysed using SILVA and DADA2 pipelines. As the first study on the faecal DNA metabarcoding of captive and wild *B. affinis*, this is the starting point to investigate the gut microbial community patterns as well as their potential roles in *B. affinis*’ health and disease developments. It is hypothesised that the current conservation status of *B. affinis* (critically endangered) might potentially be caused by some putative gut microbiomes which directly cause the population decline drastically. Thus, the outcome of this study will help us in the future conservation management and husbandry *B. affinis* towards sustainability. Furthermore, this project could provide valuable insights into the microbial community of the species.

## Materials & Methods

### Sample collections

**Figure 2:**
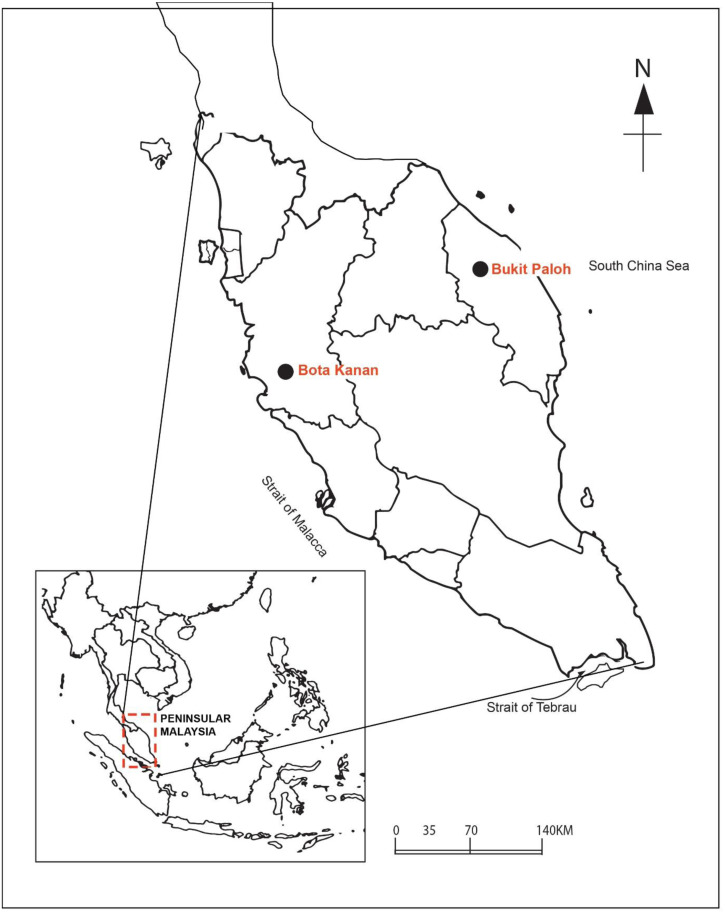
Sampling area of *B. affinis*. The faeces samples were collected from Bota Kanan, Perak (captive samples of *B. affinis*) and Bukit Paloh, Kuala Berang, Terengganu (wild samples of *B. affinis edwardmolli*).

The faecal microbial community structure from both a captive and wild population of adult *B. affinis* from the east and west coasts of peninsular Malaysia were characterised and compared (Fig. 2). The microbial community in the faeces sample was sorted and identified using standard taxonomic keys (Zemb, Achard & Hamelin, 2020). Briefly, samples were collected and transferred using a sterile spatula into a sterile 50-ml Falcon tube and stored on ice during transportation to the laboratory. The faeces samples of captive adult *B. affinis* (*N* = 5) were collected from a population at the Bota Kanan head-starting facility (BK), Perak (4.3489°N, 100.8802°E) in 2020. Meanwhile, the faeces samples of wild adult *B. affinis* (*N* = 2) were collected from a population in the Terengganu River, at Bukit Paloh, Kuala Berang (KB), Terengganu (5.0939°N, 102.7821°E) in 2021. The research and field permit approval number is B-00335-16-20, rewarded by the Department of Wildlife and Parks, peninsular Malaysia. Before DNA extraction, all faecal samples were stored at −20 °C. The 16S rRNA amplicon analysis of all faeces samples were sent to First BASE Laboratories-Apical Scientific (Malaysia).

### DNA extraction

The NucleoSpin^®^ Soil Kit (Macherey-Nagel, Germany) is commonly used to extract DNA from the soil. However, in this study, it was used to extract DNA from the faeces samples. Briefly, from the 300 mg input volume of the faeces samples, a final extraction volume of 50 µl of DNA sample was achieved and further stored at −20 °C. Purified DNA was checked for integrity on a 1% (w/v) agarose gel electrophoresis. The DNA concentration was measured using a spectrophotometer (Implen NanoPhotometer^®^ N60/N50, Germany) and fluorometric quantification using an iQuant™ Broad Range dsDNA Quantification Kit (GeneCopoeia, Inc., USA).

### 16S Illumina library and sequencing

The V3–V4 region of the 16S rRNA gene was amplified with PCR, and the primer pair 16S V3–V4 forward (5′-CCTACGGGNGGCWGCAG-3′) and 16S V3–V4 reverse (5′-GACTACHVGGGTATCTAATCC-3′) (Zhang et al., 2018). Each 50 µl of PCR mixture contained 25 µl of REDiant 2X PCR Master Mix (FirstBASE, Malaysia), 100 ng of DNA template (2.5 µl), 0.5 µ M of each primer (5 µl), and 12.5 µl of nuclease-free water. The amplification condition was as follows: an initial denaturation cycle at 95 °C for 3 min followed by 25 cycles at 95 °C for 30 s, 55 °C for 30 s, and 72 °C for 5 min. The final extension cycle at 72 °C for 10 min was also included. The 16S rRNA gene amplicons were visualised on a 1% (w/v) agarose gel electrophoresis and further purified using AMPure XP beads (BECKMAN COULTER-Life Sciences, USA) according to the manufacturer’s protocol.

The 16S rRNA gene amplicons were prepared for the Illumina MiSeq System following the 2-stage PCR protocol recommended in the Illumina 16S metagenomic library preparation instruction. With overhang adapters, the 16S rRNA gene of the targeted areas (V3–V4 region) was amplified in the first stage PCR utilising locus-specific sequence primers and overhang adapters. Forward overhang (5′-TCGTCGGCAGCGTCAGATGTGTATAA GACAG-3′) and reverse overhang (5′-GTCTCGTGGGCTCGGAGATGTGTATAAGACAG-3′) were used. All the PCR reactions were carried out with KOD-Multi & Epi^®^ (Toyobo, Japan). In the second stage of the PCR, dual guides were applied to the amplicon PCR using the Illumina Nextera XT Index Kit V2 (Illumina, USA), following the manufacturer’s instructions. The quality of the libraries was measured using the Agilent Bioanalyzer 2100 System (Agilent Technologies, San Diego, CA, USA) by the Agilent DNA 1000 Kit (Agilent Technologies, San Diego, CA, USA) and fluorometric quantification by Helixyte Green™ Quantifying Reagent (AAT Bioquest^®^, Inc., USA). According to the Illumina protocol, the libraries were normalised and pooled regarding the procedure Illumina specified and then sequenced on the MiSeq platform using 300 paired-end (PE).

### 16S rRNA metabarcoding data analysis

The area of 16S rRNA was sequenced using the PE Illumina MiSeq platform, which provides raw reads of approximately 300 bp. The forward and reverse reads were combined using QIIME2 (Caporaso et al., 2010; Lawley & Tannock, 2017). BBDuk version 39.92 has been used to remove sequence adapters, and low-quality reads from the raw reads (Bushnell, 2018). Meanwhile, QIIME2 version 2019.10 was used to align and integrate the raw readings (Bolyen et al., 2019). Finally, the Divisive Amplicon Denoising Algorithm 2 (DADA2) pipeline version 1.14 (Callahan et al., 2016; Callahan et al., 2019) was used to denoise in an attempt to remove and/or correct incorrect reads, low-quality areas, and chimeric errors to provide amplicon sequence variant (ASV) data (Nearing et al., 2018). The obtained ASV data was then employed in the subsequent steps.

The taxonomic classification was generated using the scikit-learn (Pedregosa et al., 2011) and Naive Bayes classifier (Langley, Iba & Thompson, 1992; Wang et al., 2007) against the SILVA version 132 database (Quast et al., 2013) to make individual taxonomic assignments (Callahan et al., 2019). The SILVA database was used to examine sequence similarity within ASV reads with recommended parameters at a 97% similarity level (Xue, Kable & Marco, 2018). Statistical analyses were conducted for alpha and beta diversities.

Statistical analyses were carried out in R Studio 3.6.2 using the packages phyloseq (McMurdie & Holmes, 2013), vegan (Oksanen et al., 2020), ggplot2 (Hadley, Winston & Lionel, 2019), ggrare (Kandlikar et al., 2018), and VennDiagram (Chen & Boutros, 2011; VIB-UGent, 2016). The phyloseq package tool was used to import, store, analyse, and diagrammatically show advanced phyletic sequencing information that has already been clustered into ASVs, particularly once there’s associated sample data, phylogenetic tree, and/or taxonomical assignment of the ASVs. This package leverages several tools accessible in R for ecology and phylogenetic analysis (vegan), whereas ggrare conjointly victimisation advanced/flexible graphic systems (ggplot2) to simply turn out rarefaction curve and publication-quality graphics of complex phylogenetic data. A Venn diagram is an illustration that uses circles to indicate the relationships among things or finite teams of things. Circles that overlap have a commonality, whereas circles that don’t overlap do not share those traits. Venn diagrams facilitate representing the similarities and variations between two concepts visually (McMurdie & Holmes, 2013).

A phylogenetic tree was built by combining Multiple Alignment using Fast Fourier Transform (MAFFT) (Katoh & Standley, 2013) and FastTree practises (Price, Dehal & Arkin, 2010). First, the MAFFT algorithms were used to create a multiple sequence alignment (MSA). The obtained MSA was then fed to FastTree to construct a phylogenetic tree based on maximum-likelihood nearest-neighbour interchanges (NNIs).

All sequences obtained were deposited at National Centre for Biotechnology Information (NCBI) Sequences Read Archive (SRA) databases with the BioProject accession number:  PRJNA767629 (Runs:  SAMN21919713 to  SAMN21919722).

## Results

### Sequencing results of 16S rRNA Region

**Figure 3:**
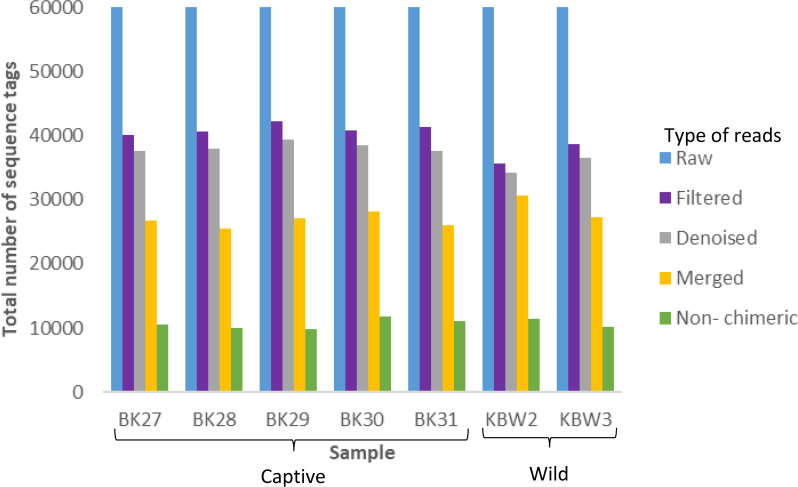
Distribution of the number of sequence tags obtained from *B. affinis* faeces samples using DADA2 pipeline. QIIME2 software version 2019.10 was used to create the bar graph for all seven samples.

A total of 420,000 bacterial 16S rRNA gene sequences were generated from seven *B. affinis* faeces samples with 60,000 each (Fig. 3). Additionally, 279,323 numbers of filtered sequences, 261,977 numbers of denoised sequences, 191,420 merged sequences, and 74,976 numbers of non-chimeric sequences tags were also analysed.

### Bacterial communities diversity

#### Alpha diversity

**Figure 4:**
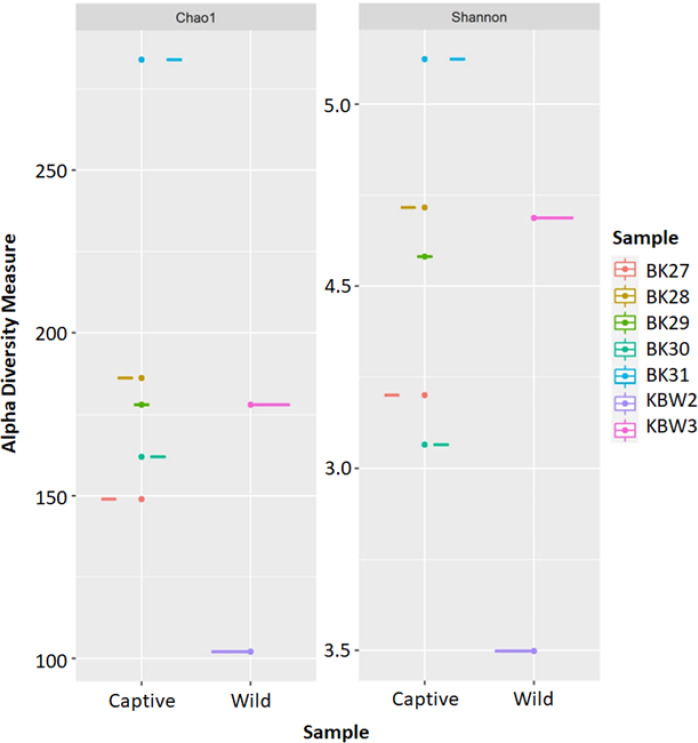
The alpha diversity analyses were inferred from 16S rRNA gene amplicon sequences for the *B. affinis*. The alpha diversity analyses were measured using Chao1 and Shannon.

**Figure 5:**
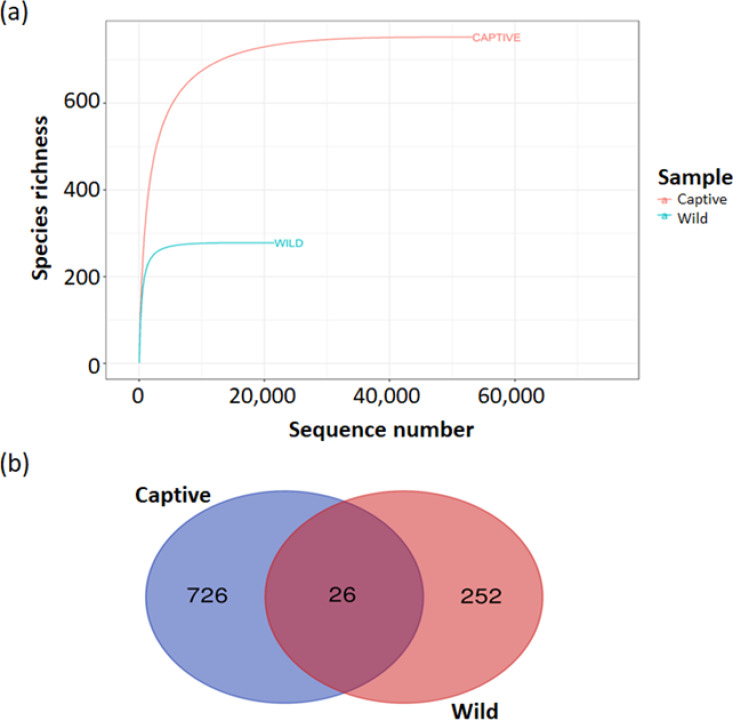
Alpha diversity analyses of bacterial communities’ determination in captive and wild *B. affinis* faeces samples. (A) Rarefaction curves showed species richness of the microbial communities in both captive and wild *B. affinis* faeces samples. (B) a Venn diagram of shared amplicon sequence variants (ASVs) between captive B. affinis faeces samples (blue) and wild *B. affinis* faeces samples (red). The ASVs was found 2.6% sequence similar between captive and wild *B. affinis* faeces samples.

The estimated ASV richness measured by the Chao1 and Shannon diversity indices varied between samples (Fig. 4). Chao1 indicated that the faeces sample, BK31, originated from captive *B. affinis,* had the highest diversity with Chao1 at 284. Conversly, the lowest was recorded from the faeces sample, KBW2, which originated from wild *B. affinis* with Chao1 at 102. The same was found for the Shannon diversity index, in which the BK31 sample showed the highest values at 5.124, whereas the lowest was for the KBW2 sample, which was determined at 3.498. To further compare the sequencing depth between captive and wild *B. affinis* faeces samples, the rarefaction curve was generated (Fig. 5A). Again, captive *B. affinis* faeces samples showed the highest sequencing depth compared to the wild *B. affinis* faeces samples.

The taxonomic distribution of the faeces microbiota was consistent between individual samples, although it differed significantly between captive and wild *B. affinis* faeces samples (Fig. 5B). In the analysed samples, a Venn diagram depicts the frequent and distinctive ASVs. Overall, captive *B. affinis* faeces samples comprised 74.9% ASVs, and wild *B. affinis* faeces samples comprised 27.7% ASVs. In captive *B. affinis* faeces samples, 72.3% of unique ASVs were found, whereas 25.1% of unique ASVs were found in wild *B. affinis* faeces samples. The overlapping portion in a Venn diagram (2.6%) represents the similar ASVs between captive and wild *B. affinis* faeces samples.

#### Beta diversity

**Figure 6:**
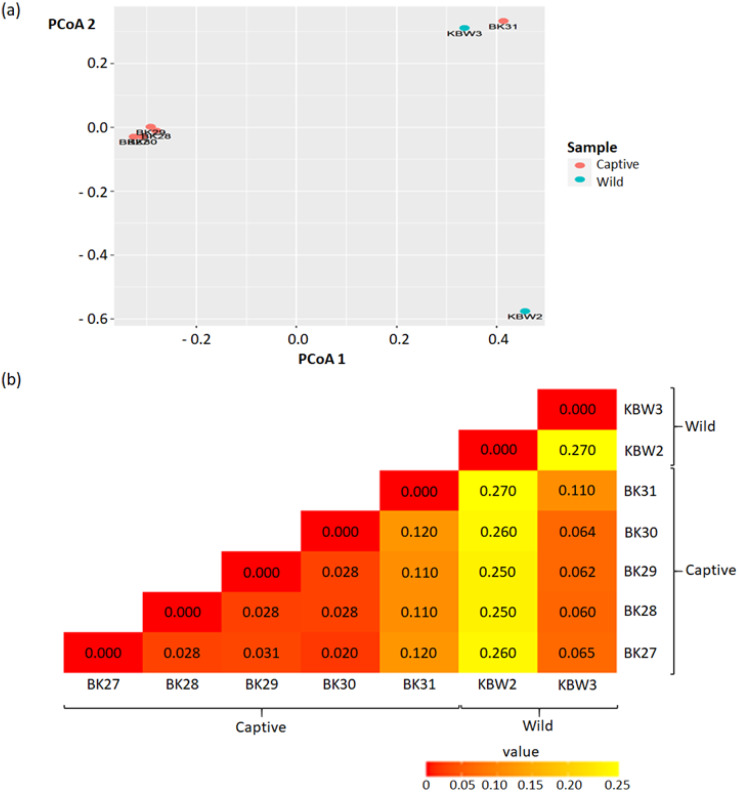
Beta diversity analyses between captive and wild *B. affinis* faeces samples. (A) The Principal Coordinates Analysis (PCoA) representations of PCoA 1 versus PCoA 2. (B) Beta diversity heatmap based on the UniFrac distance. The numbers in the grid represents the coefficient difference between all the samples.

Beta diversity was quantified using the Principal Coordinate Analysis (PCoA) analysis that quantifies the dissimilarity of ASVs (presence/absence) between captive and wild *B. affinis* faeces samples. As a result of this segmentation, PCoA analysis using the unweighted UniFrac dissimilarity index revealed that captive and wild *B. affinis* faeces samples were divided along the axis (Fig. 6A). It was feasible to observe sample clustering (28% and 32%, respectively). The wild *B. affinis* faeces samples constituted a distinct group along axes one. All samples from captive *B. affinis,* namely BK27, BK28, BK29, and BK30, were found to overlap on the two axes, except for the BK31 sample.

The beta diversity measurements are depicted in the heatmap (Fig. 6B). The numbers in the grid represent the coefficient difference between all the samples. The smaller the number of coefficient differences, the narrower the difference between the samples in terms of ASV. Overall, weighted-UniFrac distances between samples ranged between 0.00 to 0.27.

### Bacterial taxonomic profiles

**Figure 7:**
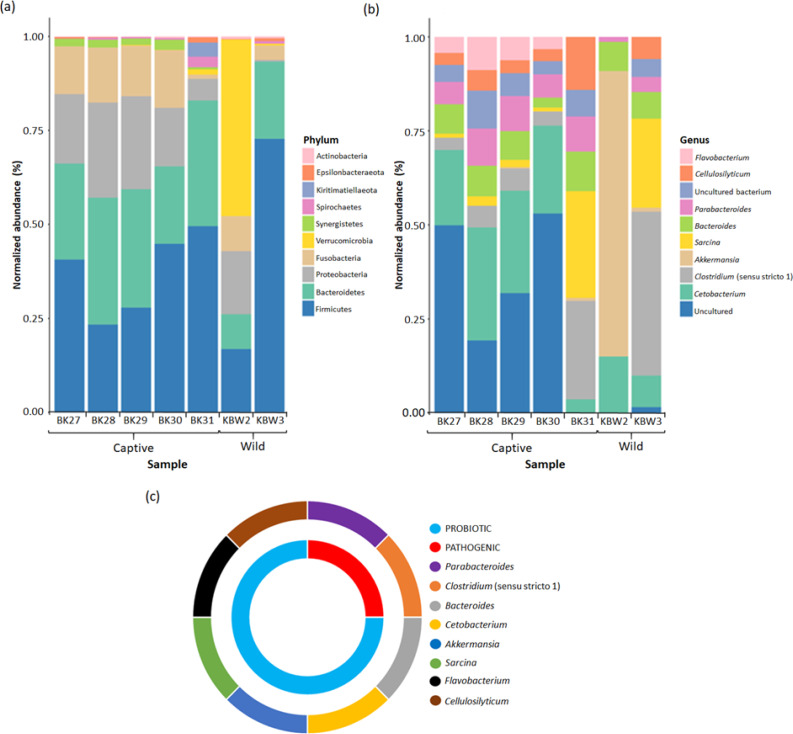
Bacterial taxonomic profiles analyses between captive and wild *B. affinis* faeces samples. The normalised abundance of the top ten genera (B) and phyla (C) present in the *B. affinis* faeces samples. (C) The classification of bacterial genera group obtained from *B. affinis* faeces samples.

The bacterial species distribution in both captive and wild *B. affinis* faeces samples was determined at the bacterial phylum and genus level (Fig. 7). The abundance of bacterial communities increases from phylum to genus in the hierarchy. It has been determined that the taxonomic makeup of 297 bacterial populations. Overall, the faeces of *B. affinis* contained 20 phyla, 28 classes, 39 orders, 70 families, and 140 bacterial genera.

In captive *B. affinis* faeces samples, three main phyla, namely Firmicutes (36.52%), Bacteroidetes (28.48%), and Proteobacteria (17.83%), were found in high abundance (Fig. 7A). Fusobacteria were found in high abundance in four captive *B. affinis* faeces samples, namely BK27 (12.58%), BK28 (14.32%), BK29 (13.35%), and BK30 (15.02%), meanwhile, low abundance in BK31(1.04%) sample. Moreover, the BK31 sample was found to be diversely distributed with various types of bacterial phyla. Nine bacterial phyla, namely Firmicutes, Bacteroidetes, Proteobacteria, Fusobacteria, Verrucomicrobia, Synergistetes, Spirochaetes, Kiritimatiellaeota, and Epsilonbacteraeota, were found in the BK31 sample. Variation was found in wild *B. affinis* faeces samples between KBW2 and KBW3 samples. In the KBW2 sample, three main phyla were found in high abundance: Verrucomicrobia (46.82%), Firmicutes (16.77%), and Proteobacteria (16.72%). In the KBW3 sample, Firmicutes (70.96%) were found in high abundance, followed by Bacteroidetes (20.04%) and Fusobacteria (3.77%). Thus, in wild *B. affinis,* three phyla were highly abundant, namely Firmicutes (44.06%), Verrucomicrobia (23.74%) and Bacteroidetes (14.70%). Overall, in all *B. affinis* faeces samples, three main phyla, namely Firmicutes (38.69%), Bacteroidetes (24.52%), and Fusobacteria (6.95%) were found in high abundance. Meanwhile, Proteobacteria (39.63%) were also detected in all samples, except for the KBW3 sample.

At the genus level, it has been found that in four captive *B. affinis* faeces samples, namely BK27 (20.06%), BK28 (30.03%), BK29 (27.25%), and BK30 (23.36%), *Cetobacterium* was found as the most abundant genus present (Fig. 7B). Furthermore, an uncultured bacterial genus was abundant in BK27, BK28, BK29, and BK30 samples. On the other hand, *Sarcina* (28.38%) and *Clostridium* (26.17%) were the most abundant genera in the BK31 sample. Similar to the phylum level, a variation in the bacterial genus was also found between KBW2 and KBW3 samples. In the KBW2 sample, only four bacteria genera were identified: *Akkermansia* (75.90%), *Cetobacterium* (15.04%), *Bacteroides* (7.75%), and *Parabacteroides* (1.31%). On the other hand, *Clostridium* (43.59%), *Sarcina* (23.65%), and *Cetobacterium* (8.41%) were found in high abundance in the KBW3 sample. Therefore, in captive *B. affinis* faeces samples, three highly abundant genera were *Cetobacterium* (20.87%), *Parabacteroides* (8.14%) *and Bacteroides* (7.34%). Meanwhile, in wild *B. affinis*, *Akkermansia* (38.49%), *Clostridium* (21.80%) and *Sarcina* (11.82%) were the three highly abundant genera.

Overall, three main genera were identified in all the faeces samples. *Cetobacterium* (67.79%) was found to be the most abundant genus, followed by *Bacteroides* (24.56%) and *Parabacteroides* (21.78%). The uncultured bacterial genus (88.51%) had the highest abundance even though not presented in BK31 and KBW2 samples. Two genera, namely *Clostridium* (35.65%) and *Sarcina* (21.48%), were found in abundance in all samples, except in the KBW2 sample.

It has been found that the majority of bacterial genera present in *B. affinis* faeces samples belong to the probiotic (75%) group, which includes *Cellulosilyticum*, *Flavobacterium*, *Sarcina*, *Akkermansia*, *Cetobacterium*, and *Bacteriodes*. In addition, potential pathogenic (25%) genera including *Parabacteroides* and *Clostridium* were also detected in the *B. affinis* faeces samples (Fig. 7C).

### Phylogenetic relationship

**Figure 8:**
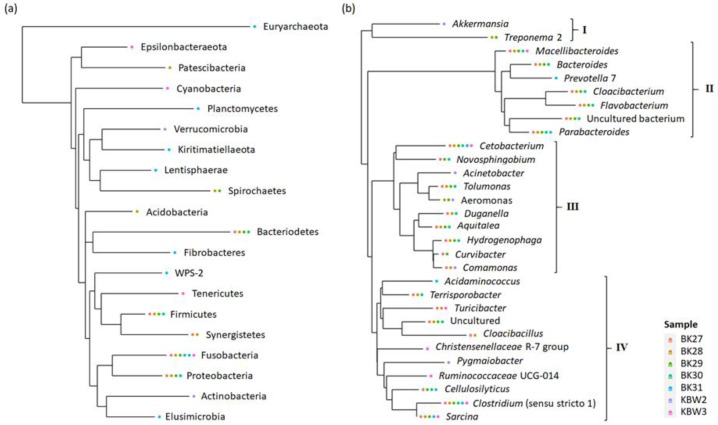
Phylogenetic relationship of bacterial community present in captive and wild *B. affinis* faeces samples. Top 30 (A) phyla and (B) genera and their abundances.

To show newly discovered phyla and genera, a phylogenetic tree has been constructed using maximum-likelihood NNIs that link the recognised phyla and genera as well as their abundances (Fig. 8). The results have suggested that the phylum tree depicts the links between the phyla present (Fig. 8A). The phylum Euryarchaeota appeared to be an outgroup. The majority of phyla displayed relationships and connections. Fusobacteria were found to be the most prevalent phylum of bacteria present in both captive and wild *B. affinis* faeces samples, followed by Bacteroidetes, Firmicutes, and Proteobacteria.

On the other hand, the genera tree depicts the four bacterial clusterings (Fig. 8B). Most of the bacterial genera in cluster 2 were found in high abundance in all faeces samples, followed by clusters 4, 3, and 1. *Cetobacterium* and *Clostridium* were highly distributed in several samples, followed by *Macellibacteroides*, *Parabacteroides*, and *Sarcina*.

## Discussion

This study used the metabarcoding approaches to analyse the bacterial population in captive and wild *B. affinis* faeces. Significant differences in bacterial populations between captive and wild groups were discovered. The results showed that the captive *B. affinis* faeces samples have a greater bacterial variety and richness than the wild *B. affinis* faeces samples. Furthermore, it was determined that most captive *B. affinis* faeces samples showed similar bacterial communities present. In contrast, wild *B. affini* s faeces samples have different bacterial communities with more significant intra-group variance. Ahasan et al. (2017) suggested that the herbivores have various microbiological requirements for accessing complex carbohydrates in the plant material they ingested as a primary food source. It is noteworthy that wild *B. affinis* are frequently regulated to a natural diet (such as molluscs and mangrove fruit) (Ahasan et al., 2017). In addition, their poor health stated by Ahasan et al. (2017) promotes the colonisation and growth of opportunistic bacteria compared to captive *B. affinis*. Hence, this might reduce the number of bacteria present in the wild *B. affinis*.

Based on the results, Firmicutes were found to dominate in all the *B. affinis* faeces samples. Bacteria from the phylum Firmicutes have been commonly found in reptiles (Hong, Wheeler & Cann, 2011) and mammals (Tsukinowa, Karita & Asano, 2008; Nelson, Apprill & Mann, 2015; Merson, Ouwerkerk & Gulino, 2014). It has been suggested that the bacteria from the phylum Firmicutes invertebrates play an essential role in helping the host gain energy and nutrients by assisting with food digestion (Wang, Cao & Li, 2016). Therefore, the frequency of Firmicutes might reflect the normal condition of the gastrointestinal tract of *B. affinis*. Furthermore, proteobacteria were also found in most of the faeces samples, except in the KBW3 sample. It is noteworthy that proteobacteria are commonly found associated with many animals, including the stranded Loggerhead sea turtles (*Caretta caretta)* (Abdelrhman, Bacci & Mancusi, 2016), as well as the Green sea turtles (*Chelonia mydas*) (Ahasan et al., 2018). It has been suggested that the high abundance of Proteobacteria in the gastrointestinal tract is a known characteristic of dysbiosis and an indicator of disease in animals (Shin, Whon & Bae, 2015). However, as a physiologically and metabolically varied group, Proteobacteria can also play a crucial role in preparing the juvenile gut for subsequent colonisation by strict anaerobes by absorbing oxygen, modifying the gut pH, as well as creating carbon dioxide and nutrients (Wilson, 2005; Chow & Lee, 2006).

In this study, the phylum Bacteroidetes was found in all the captive and wild *B. affinis* faeces samples. Bacteria belonging to the phylum Bacteroidetes are a common element of the gut microbiota of many vertebrates and turtles (Abdelrhman, Bacci & Mancusi, 2016; Nelson, Rogers & Brown, 2013; Wang, Cao & Li, 2016). It has been suggested that most bacteria belonging to the phylum Bacteroidetes carry many sets of genes encoding carbohydrate-active chemicals that help to improve the balance of the gut microbiota (Thomas, 2011; Xu et al., 2007). In humans, a high protein diet has been reported to increase the Bacteroidetes population in the gastrointestinal tract (Wu et al., 2011). As the wild *B. affinis* primarily consumes a high-protein diet such as molluscs (Moll, 1980), the results of this study showed that Bacteroidetes are highly abundant in the wild *B. affinis* samples, suggesting that the host diet directly influences the host microbiome.

Fusobacteria was another bacterial phylum abundantly discovered in captive and wild *B. affinis* faeces samples. A greater abundance of Fusobacteria has also been reported in captive seals that primarily feed on fish (Nelson, Rogers & Brown, 2013; Numberger, Herlemann & Jurgens, 2016). The typical diet in captivity on *B. affinis* is a marine fish (*Rastrelliger* sp.). Meanwhile, the wild *B. affinis* commonly consumes fish and small invertebrates that are detected to hold a high concentration of Fusobacteria (Garrity, Bell & Lilburn, 2005; Vega et l., 2009), suggesting that the prevalence of Fusobacteria in *B. affinis* faeces was influenced by the host diet. Moreover, bacteria belonging to the phyla Actinobacteria, Verrucomicrobia, and Lentisphaerae were also found in the faeces samples of captive and wild *B. affinis*. Similarly, these bacterial phyla have also been found in several animals such as loggerhead sea turtles, iguanas, dugongs, and seals (Abdelrhman, Bacci & Mancusi, 2016; Tsukinowa, Karita & Asano, 2008; Hong, Wheeler & Cann, 2011; Nelson, Rogers & Brown, 2013; Numberger, Herlemann & Jurgens, 2016).

Meanwhile, many uncultured genera have been detected in all *B. affinis* faeces samples. Microbiome analysis studies have proven the presence of many uncultured genera in freshwater fish, such as *Oreochromis niloticus* (Tsuchiya, Sakata & Sugita, 2008) and *Cyprinus carpio* (Van Kessel et al., 2011). It has also been abundantly found in the human gut (Lagier et al., 2012). It is expected that approximately 80% of the bacteria detected with molecular implements are uncultured (Turnbaugh et al., 2007; Bilen et al., 2019). Therefore, it is expected that this study will show the highest density of uncultured genera due to present taxonomical constraints (Almeida et al., 2019).

Noteworthy, the potential probiotic bacterial genera, namely *Cetobacterium*, *Bacteroides*, *Akkermansia*, *Sarcina*, *Flavobacterium*, and *Cellulosilyticum* were identified from the *B. affinis* faeces samples. *Cetobacterium* was the most abundant bacterial genus found in both captive and wild *B. affinis* faeces samples, followed by *Bacteroides*. Furthermore, both *Cetobacterium* and *Bacteroides* have been identified as frequent occupants in the guts of various aquatic mammals and fishes (Larsen, 2014; Nelson, Rogers & Brown, 2013; Roeselers, Mittge & Stephens, 2011). Interestingly, these bacterial genera have been suggested to contribute to the production of vitamin B-12 in the fish gut. For instance, high vitamin B-12 levels have been detected in the intestines of carp and tilapia (free dietary vitamin B-12) colonised with *Cetobacterium* and *Bacteroides* (Sugita, Miyajima & Deguchi, 1991).

Besides, bacteria belonging to the genus *Akkermansia* have been suggested to contribute to the stimulation and manipulation of gut immune responses (Chen et al., 2019; Cekanaviciute et al., 2017; Rothhammer, Borucki & Tjon, 2018; Rojas et al., 2019). Moreover, *Sarcina*, *Flavobacterium*, and *Cellulosilyticum* have all been suggested to contributing to host food digestion. Due to their ability to secrete a variety of extracellular hydrolytic enzymes and to breakdown complex carbohydrates such as cellulose, hemicellulose, and lignocellulose (Ahasan et al., 2018; Ilker & Ogram, 2006; McKnight et al., 2020; Cai & Dong, 2010; Li et al., 2011).

In this study, the presence of potentially pathogenic bacterial genera has also been detected. *Clostridium* was found in all *B. affinis* faeces samples except the KBW2 sample. Bacterial species from the genus *Clostridium,* such as *Clostridium botulinum,* have been reported as pathogens due to their ability to produce botulinum. This neurotoxin causes botulism in both animals and humans (Cherington, 2004). Moreover, another potential pathogenic bacterial genus, namely *Parabacteroides,* has also been identified. Although *Parabacteroides* is commonly associated with the gastrointestinal tract of animals, *Parabacteroides* have also been suggested as commensal bacteria (Sakamoto & Benno, 2006; Allsop & Stickler, 1985; Ezeji et al., 2021). However, some studies have reported that *Parabacteroides* can be identified as pathogens (Kverka et al., 2011; Rodriguez-Palacios et al., 2020). In this study, *Parabacteroides* has been found in both captive and wild *B. affinis* faeces samples. The presentation suggests a host-pathogen interaction that could be studied.

## Conclusions

In summary, this study described the faecal bacterial populations of captive and wild critically endangered Southern River Terrapins, *B. affinis*. Our results indicated that the captive *B. affinis* faeces samples have a greater bacterial variety and richness than wild *B. affinis* faeces samples. Therefore, we propose the application of some pharmaceuticals for disease treatments and combat any potential opportunistic bacterial related infections, for routine conservation management of *B. affinis*. However, the approach cannot be considered a substitute for the ever-important practice of animal husbandry of the captive population. In addition, daily observation and good record keeping of *B. affinis* behaviour and feeding activity enables early discovery of abnormalities, allowing for a diagnosis to be made before the majority of the captive population gets ill. If treatment is recommended, it will be most effective if started early in the disease while the *B. affinis* are still in good health.

As currently there is limited information on the gut microbiota of *B. affinis*, the faecal bacterial populations are hoped to provide a basis for further studies of *B. affinis*’ gut microbiota. A research gap that is still required would be on the knowledge gap on harmful microorganisms such as viruses, bacteria, parasites, and fungus that has not yet been investigated on the subject matter, which has the potential to spread among or between hosts. Aside from that, future research could look into the impact of the dominant phylum (Proteobacteria) and genus (*Cetobacterium*). Thus, the presence of potentially pathogenic genera (*Clostridium* and *Parabacteroides*) on the health and productivity of *B. affinis*, assisting us in developing a long-term management and conservation strategy for *B. affinis* towards sustainability.

## Additional Information and Declarations

### Field Study Permissions

The following information was supplied relating to field study approvals (i.e., approving body and any reference numbers): Department of Wildlife and National Parks, Peninsular Malaysia was permitted B-00335-16-20 for this study.

### DNA Deposition

The following information was supplied regarding the deposition of DNA sequences: The sequences are available at NCBI: PRJNA767629.

### Data Availability

The following information was supplied regarding data availability: All sequences are available at NCBI SRA: PRJNA767629 (Runs: SAMN21919713 to SAMN21919722).
